# Supplementary material for: Short-term health effects of an urban regeneration programme in deprived neighbourhoods of Barcelona
Source: PLoS One. 2024 Apr 17;19(4):e0300470. doi: 10.1371/journal.pone.0300470 (PMC11023398; doi:10.1371/journal.pone.0300470)
Supplement: S1 File — (DOCX) [file pone.0300470.s001.docx]

**Supporting information**

**S1 Table 1. Characteristics of the study population stratified by sex and group: weighted data by the inverse of the propensity score. Barcelona, 2016 and 2021^a^.**

|  | **2016** | | | | | | **2021** | | | | | |
| --- | --- | --- | --- | --- | --- | --- | --- | --- | --- | --- | --- | --- |
|  | **Men** | | | **Women** | | | **Men** | | | **Women** | | |
|  | **Compa-rison**  **N=304** | **Moderate**  **intensity**  **N=291** | **High**  **intensity**  **N=310** | **Compa-rison**  **N=324** | **Moderate**  **intensity**  **N=318** | **High**  **intensity**  **N=329** | **Compa-rison**  **N=319** | **Moderate**  **intensity**  **N=297** | **High**  **intensity**  **N=310** | **Compa-rison**  **N=321** | **Moderate**  **intensity**  **N=301** | **High**  **intensity**  **N=315** |
| **Age** | | | | | | | | | | | | |
| 15-34 | 23.4 | 23.3 | 26.4 | 27.2 | 23.8 | 25.2 | 25.7 | 25.9 | 25.2 | 25.2 | 24.8 | 25.2 |
| 35-64 | 55.7 | 54.9 | 53.6 | 45.6 | 47.2 | 47.1 | 53.2 | 54.5 | 54.7 | 47.9 | 48.2 | 47.9 |
| +65 | 20.9 | 21.8 | 20.0 | 27.2 | 29.0 | 27.7 | 21.1 | 19.6 | 20.1 | 26.9 | 27.0 | 26.9 |
| p-value**^b^** | 0.971 |  |  | 0.978 |  |  | 0.997 |  |  | 0.999 |  |  |
| **Occupational social class** | | | | | | | | | | | | |
| Non-manual | 16.7 | 15.7 | 16.7 | 16.2 | 14.4 | 15.6 | 15.3 | 15.7 | 16.3 | 16.1 | 14.9 | 19.5 |
| Manual | 83.3 | 84.3 | 83.3 | 83.8 | 85.6 | 84.4 | 84.7 | 84.3 | 83.7 | 83.9 | 85.1 | 80.5 |
| Missing | 0 | 0 | 0 | 0 | 0 | 0 | 0 | 0 | 0 | 0 | 0 | 0 |
| p-value **^b^** | 0.955 |  |  | 0.902 |  |  | 0.953 |  |  | 0.637 |  |  |
| **Employment situation** | | | | | | | | | | | | |
| Employed | 55.9 | 56.6 | 54.1 | 48.3 | 46.2 | 45.5 | 54.6 | 53.0 | 55.9 | 49.6 | 47.8 | 48.0 |
| Unemployed | 9.5 | 9.3 | 11.0 | 8.5 | 10.6 | 7.7 | 9.5 | 11.7 | 10.7 | 8.4 | 8.9 | 9.1 |
| Others | 34.6 | 34.1 | 34.9 | 43.2 | 43.2 | 46.8 | 35.9 | 35.3 | 33.4 | 42.0 | 43.3 | 42.9 |
| Missing | 0 | 0 | 0 | 0 | 0 | 0 |  |  |  | 0 | 0 | 0 |
| p-value **^b^** | 0.969 |  |  | 0.885 |  |  | 0.967 |  |  | 0.995 |  |  |
| **Country of origin** |  |  |  |  |  |  |  |  |  |  |  |  |
| Autochthonous | 72.0 | 75.1 | 70.0 | 72.8 | 75.4 | 71.3 | 73.7 | 73.9 | 70.4 | 74.2 | 74.1 | 72.3 |
| Foreign born | 28.0 | 24.9 | 30.0 | 27.2 | 24.6 | 28.7 | 26.3 | 26.1 | 29.6 | 25.8 | 25.9 | 27.7 |
| Missing | 0 | 0 | 0 | 0 | 0 | 0 |  |  |  | 0 | 0 | 0 |
| p-value **^b^** | 0.710 |  |  | 0.725 |  |  | 0.744 |  |  | 0.901 |  |  |
| **Population turnover** | | | | | | | | | | | | |
| ≤6 years | 18.8 | 14.8 | 21.9 | 15.5 | 12.1 | 11.5 | 21.5 | 21.1 | 25.9 | 21.1 | 17.2 | 18.6 |
| >6 years | 80.8 | 83.9 | 77.5 | 84.5 | 87.9 | 88.5 | 78.0 | 78.9 | 74.1 | 78.9 | 81.1 | 81.4 |
| Missing | 0.4 | 1.3 | 0.6 | 0 | 0 | 0 | 0.5 | 0 | 0 | 0 | 1.7 | 0 |
| p-value **^b^** | 0.541 |  |  | 0.559 |  |  | 0.612 |  |  | 0.711 |  |  |

**^a^** weighted data by the inverse of the propensity score; re-weighting the data implies changes in the original sample size in control and intervention groups.

**^b^** p-values of significance across comparison and intervention type.

**S1 Table 2. Change in education level by group between 2016 and 2021 in Barcelona**^a^**.**

|  | **Comparison** | | | **Moderate intensity** | | | **High intensity** | | |
| --- | --- | --- | --- | --- | --- | --- | --- | --- | --- |
|  | **2016**  **%** | **2021**  **%** | **Change**  **%** | **2016**  **%** | **2021**  **%** | **Change**  **%** | **2016**  **%** | **2021**  **%** | **Change**  **%** |
| **Incomplete**  **Primary or less** | 5.2 | 2.6 | -2.6 | 4.6 | 2.2 | -2.4 | 5.8 | 2.7 | -3.1 |
| **Competed primary** | 25.2 | 20.7 | -4.5 | 30.3 | 25.7 | -4.6 | 32.4 | 27.7 | -4.7 |
| **Low secondary** | 26.2 | 27.7 | 1.5 | 26.5 | 29.5 | 3.0 | 31.4 | 33.8 | -2.4 |
| **High secondary** | 23.2 | 24.9 | 1.7 | 20.5 | 21.9 | 1.4 | 19.2 | 22.2 | -3.0 |
| **University** | 20.2 | 24.2 | 4.0 | 18.1 | 20.7 | 2.6 | 11.2 | 13.6 | 2.4 |

^a^The Table shows the differences in the distribution of the education level between 2016 and 2021 by comparison and intervention groups. The proportion of lower education decreases while higher education increases for all groups, without differences.

**S1 Table 3. DiD and significance of health and health-related behaviour between comparison and intervention groups stratified by sex. Barcelona, 2016 and 2021. Comparison group includes 28 neighbourhood of low and middle income.**

|  | Type of  intervention | Men | Women |
| --- | --- | --- | --- |
|  |  | DiD^a^  (p-value) | DiD^a^  (p-value) |
| **Poor mental health** | Comparison | - | - |
|  | Moderate | 5.9 (0.476) | -10.7 (0.082) |
|  | High | 6.1 (0.478) | **-16.8 (0.005)** |
| **Psychotropic drug use** | Comparison | - | - |
|  | Moderate | 8.2 (0.108) | 0.04 (0.946) |
|  | High | **9.8 (0.043)** | -12.8 (0.209) |
| **Alcohol abuse** | Comparison | - | - |
|  | Moderate | -1.1 (0.771) | 4.2 (0.102) |
|  | High | -5.7 (0.430) | 0.5 (0.889) |
| **Poor self-perceived health** | Comparison | - | - |
|  | Moderate | 8.2 (0.318) | -1.2 (0.796) |
|  | High | -0.8 (0.863) | **-21.9 (0.005)** |
| **Moderate and intense**  **physical activity** | Comparison |  | - |
|  | Moderate | 0.4 (0.959) | 5.8 (0.264) |
|  | High | 5.6 (0.650) | **12.3 (0.047)** |
| **Obesity** | Comparison | - | - |
|  | Moderate | -1.5 (0.806) | -6.3 (0.213) |
|  | High | -7.7 (0.301) | -1.4 (0.801) |

^a^difference-in-difference and p-value estimated by linear regressions and adjusted by age; weighted data by the inverse of the propensity score
